# Toughening Thermoelectric Materials: From Mechanisms to Applications

**DOI:** 10.3390/ijms24076325

**Published:** 2023-03-28

**Authors:** Luoqi Wu, Xiaobin Feng, Ke Cao, Guodong Li

**Affiliations:** 1 Hubei Key Laboratory of Theory and Application of Advanced Materials Mechanics, Wuhan University of Technology, Wuhan 430070, China; 2 School of Mechano-Electronic Engineering, Xidian University, Xi’an 710071, China; caoke@xidian.edu.cn; 3 State Key Laboratory of Advanced Technology for Materials Synthesis and Processing, Wuhan University of Technology, Wuhan 430070, China

**Keywords:** thermoelectric materials, ductile, toughening mechanisms, flexible thermoelectric devices

## Abstract

With the tendency of thermoelectric semiconductor devices towards miniaturization, integration, and flexibility, there is an urgent need to develop high-performance thermoelectric materials. Compared with the continuously enhanced thermoelectric properties of thermoelectric materials, the understanding of toughening mechanisms lags behind. Recent advances in thermoelectric materials with novel crystal structures show intrinsic ductility. In addition, some promising toughening strategies provide new opportunities for further improving the mechanical strength and ductility of thermoelectric materials. The synergistic mechanisms between microstructure-mechanical performances are expected to show a large set of potential applications in flexible thermoelectric devices. This review explores enlightening research into recent intrinsically ductile thermoelectric materials and promising toughening strategies of thermoelectric materials to elucidate their applications in the field of flexible thermoelectric devices.

## 1. Introduction

Since the 21st century, the over-exploitation of fossil fuels has aggravated the energy crisis and environmental pollution. Therefore, it is urgent to pursue renewable energy instead of traditional energy. Thermoelectric materials and devices can directly convert waste heat into electricity, which is a cutting-edge technology for traditional energy conservation and replacement. Benefiting from reliable, silent, and eco-friendly [1] merits, thermoelectric devices have been widely applied in aerospace, biomedicine, and integrated circuit [2,3,4,5]. In the investigation of the origin of thermoelectric performances, thermoelectric effects include the Seebeck effect and the Peltier effect [6]. The Seebeck effect refers to the connection of two kinds of conductors or semiconductors to form a loop, where the loop current exists due to the thermoelectricity power caused by the joint of two ends with different temperatures Δ*T*. In contrast, the Peltier effect is interpreted as the heat absorbed and released at the junction when the current is inputted in the loop consisting of two different materials. Thermoelectric effects are the theoretical foundation of the thermoelectric generator and semiconductor refrigeration. In addition, the energy conversion efficiency of thermoelectric materials is related to the thermoelectric figure of merit (*zT*), which is a dimensionless quantity reflecting the thermoelectric performance [7,8], and can be calculated as follows: $zT=S^{2}\sigma T/\kappa$, where *S*, *σ*, *T*, *κ* are the Seebeck coefficient, electrical conductivity, absolute temperature, and thermal conductivity, respectively [9].

In recent years, significant progress has been made in the *zT* value of thermoelectric materials, such as Bi_2_Te_3_ [10], filled skutterudite compounds [11,12], and half-Heusler compounds [13]. For example, the nanolayered p-type Bi_2_Te_3_ prepared by spark plasma sintering shows a maximum *zT* of 1.35 at 300 K [10]. The multiple-Filled Skutterudites CoSb_3_ realizes *zT*= 1.7 at 850 K. By the past few years, novel thermoelectric materials with higher thermoelectric performance were developed. For instance, the *zT* value of the Cu_2_Se compound reaches 1.5 at a high temperature of 1000 K. The Cu_2_Se system exhibits fast ionic features with extremely low lattice thermal conductivity, which is due to Cu ions flowing in a highly disordered manner in the Se sublattice [14]. Based on this phenomenon, a new concept of “phonon-liquid electron-crystal” was proposed. The ultra-high *zT* of these newly-surged thermoelectric materials mainly benefit from the “quasi-liquid” mobility of Cu ions. Similarly, copper/silver-based fast ionic conductors, such as Cu_2_S, Cu_2_Te, Ag_2_Se, Ag_2_S, and Ag_2_Te thermoelectric compounds, have been reported to exhibit superior thermoelectric properties and potential applications [15,16,17]. Traditional strategies for enhancing *zT* include band convergence [18], resonance energy states [19], doping [20], and solid solution [21]. For example, a series of high-performance ternary Ag_2_Se_1−x_Te_x_ (*x* = 0.1, 0.2, 0.3, 0.4, and 0.5) near room-temperature thermoelectric materials were prepared by mixing the Te solute into Ag_2_Se matrix, resulting in a high *zT* value greater than 1 [22]. The *zT* of Sn_0.83_Cd_0.05_Sb_0.12_Te alloy is increased to 1.1 at 823 K by doping Sb in SnTe-CdTe alloy to regulate the vacancy and band structure [23]. In addition, the density functional theory (DFT) simulation on Mg_2_Si shows that a novel twin boundary strategy endows a decreased lattice thermal conductivity by ~90% without significantly degrading electrical properties [24]. Similarly, doping S into the *p*-type S_y_Co_4_Sb_12-2y_S_2y_ prepared by high-pressure high-temperature (HPHT) method was proved to not change the electrical conductivities of pristine counterpart, while the Seebeck coefficient of S_0.10_Co_4_Sb_11.80_S_0.20_ was remarkably increased by 60.6% compared with Co_4_Sb_12_ [25]. In addition, to reveal the origin of decreased lattice thermal conductivity, Raman scattering spectroscopy was performed on S_y_Co_4−x_Ni_x_Sb_12_, showing dopant S and Ni were at co-sites, leading to the highest *zT* of 0.81 at 800 K [26]. Porous AgS was prepared by a novel high-pressure method with reduced temperature (573 K) and a shorter time (10 min), showing the highest *zT* value of 0.62 at 560 K [27].

However, compared with continuously enhanced thermoelectric properties, the mechanical properties of thermoelectric materials have considerably lagged behind. Especially the lack of ductility of inorganic thermoelectric semiconductors severely hinders their commercial applications due to their intrinsic ionic, covalent, and/or van der Waals bonds [28]. Most thermoelectric semiconductors fracture immediately after the linear elasticity stage at room-temperature mechanical testing, indicating a typical catastrophic brittle failure. For example, the indentation-crack technique and uniaxial compression loading were employed to evaluate the mechanical properties of Cu_2_Se prepared by spark plasma sintering. The fracture toughness, compressive strength, and plastic strain of Cu_2_Se were measured to be merely about 2 MPa·m^1/2^, 45 MPa, and 3%, respectively [29]. Another example is the typical Bi_2_Te_3_ compound, the crystal structure of which consists of a five-layer substructure following the sequence Te1–Bi–Te2–Bi–Te1. Note that the number refers to different types of Te–Bi bonds and two neighboring substructures connected by two Te1 layers, which bond with weak van der Waals bonds, resulting in a brittle cleavage along the (*00l*) axis. [30]. Furthermore, the strength of high-textured Bi_2_Te_3_-based materials synthesized by the thermal explosion and solvothermal techniques is improved significantly with degrading in ductility [31,32]. In addition, GeTe-based materials exhibit a creep distortion of over 7% at high temperatures and an engineering compressive strain of ~1%, strongly impeding their applications at a temperature range from 600K to 900K [33]. Besides, although the toughness of SnSe is improved by solvothermal synthesis, its fracture strain is only 0.02%, much lower than the commercial requirements [34]. The intrinsic brittle originates from the chemical Sn-Sn bonds along the *b-c* plane are much stronger than Sn–Sn bonds along the *a*-direction, and thus, one is prone to cleavage fracture because of the weak Sn–Se between *b-c* planes [35]. Therefore, it is of great significance to develop ductile thermoelectric materials for improved reliability and service life of flexible thermoelectric devices. This review will first elucidate the intrinsically ductile thermoelectric materials. It also provides a better understanding of toughening strategies of thermoelectric materials and explores their potential applications on flexible thermoelectric devices.

## 2. Intrinsically Ductile Thermoelectric Materials

A ductile material needs to satisfy two conditions. One is a comparatively low slipping barrier for slip planes to guarantee atoms, dislocations, or interfaces that can easily move along these planes under an external force. The other is some comparatively strong interaction between the atoms in these slip planes to ensure these planes remain stable to maintain an intact material during the slip process. The intrinsic brittleness feature of thermoelectric materials hinders their applications. Recent studies revealed intrinsically ductile thermoelectric materials with novel crystal structures.

**Figure 1:**
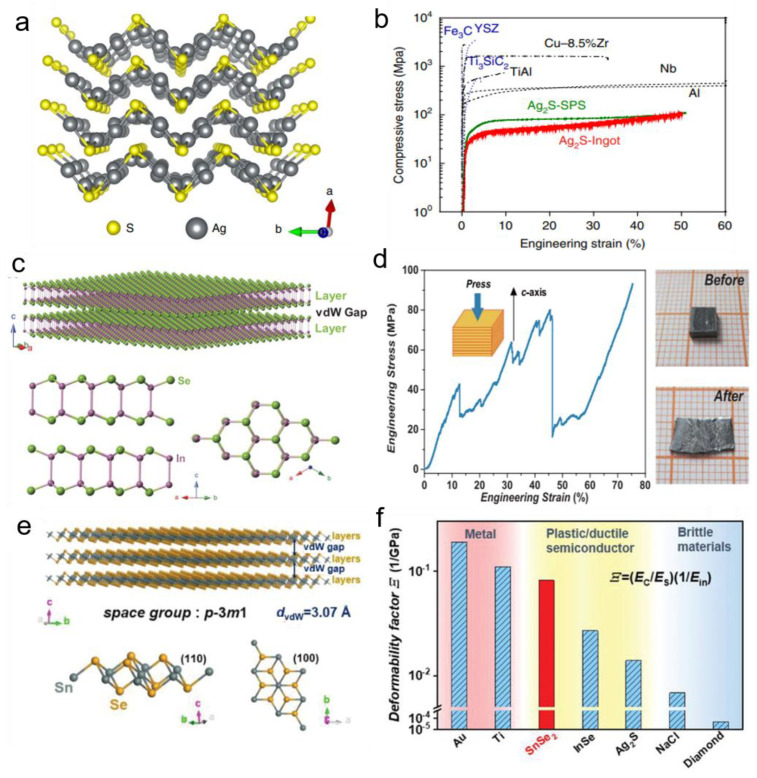
The intrinsically ductile thermoelectric materials. (**a**) A perspective view of the α–Ag_2_S crystal structure along the [001] direction [17]. (**b**) The compressive stress-strain curve exhibits outstanding ductility of *α–*Ag_2_S (Reprinted with permission from ref [17]. Copyright 2018 Springer Nature). (**c**) The crystal structure of *β–*InSe [38]. (**d**) The compressive stress-strain curve of InSe deformed along the *c*-axis and images of samples before and after compression (Reprinted with permission from ref [38]. Copyright 2018 American Association for the Advancement of Science). (**e**) The crystal structure of SnSe_2_ [41]. (**f**) The deformability factors (Σ) of ductile semiconductors, metals, and brittle materials (Reprinted from ref [41] under the terms of a Creative Commons CC BY License. Published 2022 Wiley-VCH GmbH).

As shown in Figure 1a, the atom structure of *α–*Ag_2_S is folded layers stacked along the a-axis at room temperature. Figure 1b shows an exceptional tensile, compression, and three-point bending strain of 4.2%, over 50%, and 20%, respectively [17]. To explore the mechanism of outstanding mechanical properties in *α–*Ag_2_S, DFT was employed to simulate the slipping processes by dividing the slip within one crystallographic period into 12 steps to figure out its structural and chemical bonding feature on the atomistic level. From the results of the total energy of each step, there is a low slipping energy barrier Δ*E_S_* and a large cleavage energy Δ*E_C_* in *α–*Ag_2_S analogous to that of Titanium and Magnesium [36]. Consequently, *α–*Ag_2_S exhibits extraordinary metal-like ductility. Furthermore, quantum chemical techniques in position space were applied to investigate the performance of the chemical bonding, revealing that a large Δ*E_C_* is caused by the irregularity of silver atoms producing additional Ag–S and Ag–Ag bonds. Besides, in the “zigzag” layered structure, the bonds between two layers are formed by the S atoms in the upper layer and Ag atoms in the lower layer. S atoms can smoothly break bonds with Ag and bond with another Ag in the groove consisting of six Ag atoms, leading to a large plastic deformation without cleaving or cracking. To fully understand the deformation mechanism of *α–*Ag_2_S, ab-initio-based density function theory was applied to analyze the response of the bonds and atomic patterns under pure shear load [37]. The Ag–S octagon framework steadily compacts or expands with a bond angle bent from 76.5° to 148.6° until this framework is destroyed by bond breakage, which clearly shows that the bond angle leads the plastic mechanism for pure shearing along (001)[010]. In the process of shearing along (100)[010], the lengths of Ag–S bonds slightly extend or contract, and a newly formed metallic bond reinforces structure and coupling with the Ag–S framework to hinder further slip. Under pure shear strain, bond angles and bond lengths keep Ag–S octagon framework integrity without bonds breaking, which contributes to the plastic deformation of *α–*Ag_2_S.

Large plasticity was also observed in van der Waals layered InSe. Figure 1c exhibits the crystal structure of InSe, adopting a layered structure with In-Se hexagonal lattice in the *ab* place and atom sequence of Se–In–In–Se in the *c*-axis. Bulk polycrystal InSe is intrinsic brittleness, but surprisingly, Figure 1d reveals bulk InSe with single-crystalline structure exhibits ~80% compressive strain along the *c*-axis, ~12% elongation perpendicular to the c-axis, and can be changed to various shapes at room temperature [38]. To elucidate the interactions of intra- and interlayers, the elastic stiffness constants (c_ij_) were used to deduce intralayer Young’s modulus *E*_in,_ and DFT was invoked to inspect the interlayer interactions and relative glide process. From the former, the value of *E*_in_ is ~53 GPa, which is the lowest among the hexagonal structure and indicates the intralayer bond rigidity and interlayer interactions contribute less to the flexibility of mono- or few-layer InSe. Then, through the simulation of DFT, the *E_S_* is as low as 0.058 eV per atom. By contrast, the *E_C_* is 0.084 eV per atom, which is significantly higher than brittle materials. For the intralayer interactions, the electron localization function (ELF) of the In–Se bond is as low as that of metals [39], leading to a low bond rigidity and intralayer modulus. Besides, the In–In bond shows a high ELF, indicating a localized covalent feature. For the interlayer, from the charge density distortion, there is a long-range coulombic In–Se bond between layers and the Se–Se-like van der Waals force with a large figure of *E_c_* to ensure structural integrity, during interlayer gliding and cross-layer dislocation slip. More importantly, a deformability factor was proposed to select other candidate plastic bulk materials, $\Sigma =\left(E_{c}/E_{s}\right)\left(1/E_{in}\right)$, where $E_{c}$, $E_{s}$, and $E_{in}$ are the cleavage energy, slipping energy, and the in-plane Young’s modulus along the slip direction, respectively, which is in good agreement with early literature [40]. Based on this rule, an important deformability factor is the existence of bulk 2D vdW single-crystalline SnSe_2_ (Figure 1e), which endures a large bending strain as high as 15% in a three-point bending test without cracking and retains good integrity on the microscopic scale [41]. The calculated $E_{s}$ along (001)<120> is 0.022 eV per atom, lower than InSe along (001)[100] and Ag_2_S along (100)[001], while the $E_{c}$ along (001) is 0.143 eV per atom, higher than InSe and Ag_2_S. Therefore, Σ is much higher than those of ductile InSe and Ag_2_S, as visualized in Figure 1f.

## 3. Toughening Strategies of Thermoelectric Materials

### 3.1. Size Effect

Inorganic thermoelectric materials are generally weak and brittle on a macroscopic scale. However, when the feature size reduces to the micron/nanometer scale, the mechanical properties of small-sized materials are quite different from that of bulks. There is a size effect on mechanical properties at the micro-/nanoscale, namely, the smaller, the stronger [42,43]. However, when grain sizes decrease into the nanocrystalline regime, the limited ductility/deformability becomes the Achilles’ heel of these ultrastrong nanocrystals [44]. For example, the hardness of the hot-pressed PbTe-PbS with finer grain size is 1.18 ± 0.09 GPa, which is about 70% higher than that of the cast sample (0.68 ± 0.07 GPa) [45]. The Bi-Sb-Te samples with micro-/nano-sized grain were fabricated by different processes, such as zone-melting (ZM), hot-press, and spark plasma sintering (SPS). Among them, the strengths of samples with smaller grain sizes prepared by hot-press and SPS are about 4 times and 10 times higher than that of ZM specimens (30 MPa), respectively [46]. Another example is the nanostructure α-MgAgSb thermoelectric materials with a grain size of about 150 nm prepared by ball milling, and hot press process exhibits a significantly high hardness and strength of 3.3 GPa and 389.6 MPa, respectively [47]. By melt spinning and plasma-activated sintering (MS-PAS) method, the compressive strength of BiSbTe alloys increases with decreased grain size due to grain refinement [48].

**Figure 2:**
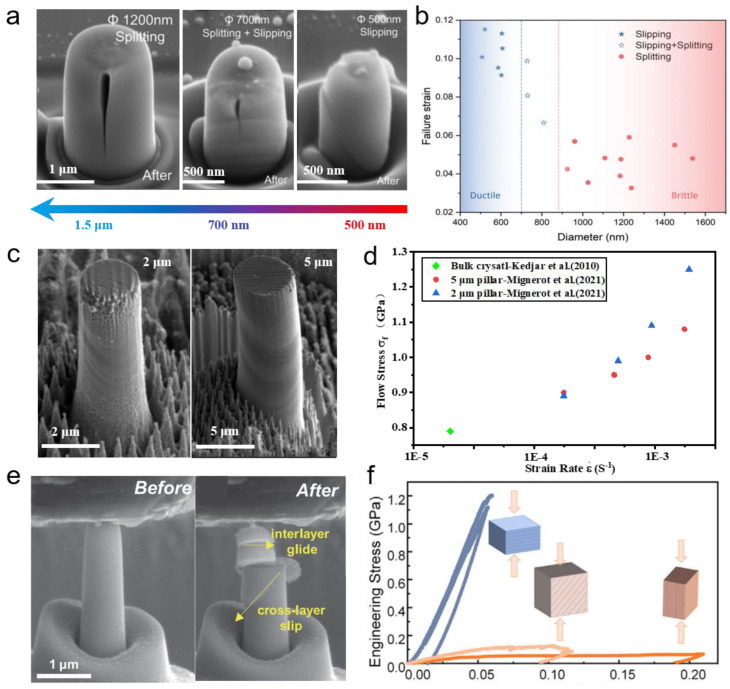
Ductile thermoelectric materials at the micro-/nanoscale (**a**) SEM images of GaN pillars with different diameters after uniaxial compression along [0001] direction [50]. (**b**) Size effect on brittle-ductile transition in GaN deformation (Reprinted with permission from ref [50]. Copyright 2020 Royal Society of Chemistry). (**c**) SEM images of InSb pillars with a diameter of 2 μm and 5 μm after compression along <123> direction [51]. (**d**) Flow stress with different strain stress for 2 μm and 5 μm InSb pillars and compared to bulk crystal (Reprinted from ref [51] under the terms of a Creative Commons CC BY License. Publish 2021 Springer Nature). (**e**) SEM image of the InSe micropillars along the *c*-axis before and after compression. (Reprinted from ref [36] under the terms of a Creative Commons CC BY License. Publish 2022 Wiley-VCH GmbH) (**f**) The engineering stress-strain curves for the compression tests on GaSe pillars with different orientations (Reprinted with permission from ref [52]. Copyright 2022 Elsevier).

In addition to the internal size, the sample size (or the external size) also shows a great impact on mechanical properties. Micro-nanomechanical tests, e.g., nanoindentation and micro-compression, have been employed to investigate the plastic deformation and failure mechanism of inorganic thermoelectric materials at micro-/nanoscale [49]. Generally, the lack of plastic deformation capacity in bulk GaN limits its applications. However, as shown in Figure 2b, a transition from brittle to ductile with the failure strain increases from ~3 % to ~12 % was observed when the diameter of pillars reduced from 1.5 μm to 0.4 μm [50]. From Scanning Electron Microscope (SEM) and Transmission Electron Microscope (TEM) observations, it shows clearly that the crack nucleation/propagation at the microscale, slipping at the intermediate sizes, and slipping without a crack when the pillar diameter is below 0.7 μm. Figure 2c shows the existence of twins found in deformed InSb micropillars, which had never been reported in bulk InSb before [51]. Figure 2d depicts the flow stress of the micropillar is significantly greater than that of the bulk, showing the smaller the diameter of the pillar, the stronger. The orientation-dependent plastic deformation was observed in single-crystal GaSe by in-situ SEM compression test, as shown in Figure 2f [52]. When the load is along, slanted at an angle of 45°, and perpendicular to the *c*-axis, the GaSe pillars exhibit compressive strains of ~6.0%, ~9.3%, and ~20.2%, respectively. The low slip energy and high cleavage energy are calculated by DFT, indicating that easy interlayer gliding without fracture is the cause of large plasticity in GaSe at small scales. A similar phenomenon is sketched in Figure 2e in that the cross-layer slip and interlayer gliding of InSe pillars lead to a strain burst along *c*-axis [38]. Nanoindentation tests can also be used to explore temperature and strain rate effects on mechanical properties [53,54,55]. Both theoretical calculations and indentation tests on thermoelectric materials at micro-/nanoscale reveal a ductile-brittle transition from perfect dislocations to partial dislocations. Therefore, the mechanical properties of thermoelectric materials can be optimized by size effect for structural and functional applications.

### 3.2. Twin Boundary Engineering

**Figure 3:**
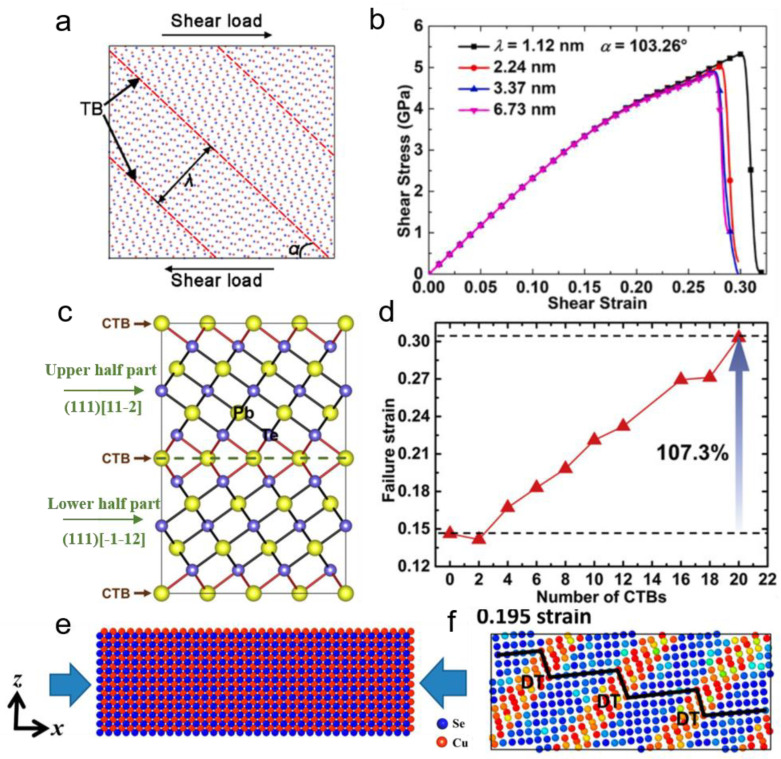
Twin boundary engineering on mechanical properties of thermoelectric materials. (**a**) The atomic model under shear load with TB spacing *λ* and orientation [60]. (**b**) The ideal strength increases with decreasing the spacing of TB (Reprinted with permission from ref [60], Copyright 2022 Elsevier). (**c**) The atomic structure of nanotwinned PbTe [61]. (**d**) The ductile enhanced with the number of CTBs increases (Reprinted with permission from ref [61]. Copyright 2022 Elsevier). (**e**) MD model of *β*-Cu_2_Se under uniaxial compression [62]. (**f**) At 1000 K, the existence of the deformation twin changes the deformation mode into a combination of compression and shear conditions (Reprinted with permission from ref [62]. Copyright 2022 American Chemical Society).

Twin boundary engineering is another effective strategy to enhance the mechanical properties of thermoelectric materials [56]. The twin boundary (TB) minimizes the boundary spacing to the nanoscale and hinders the dislocation movement to impact the mechanical properties. For instance, nanotwins in Bi_2_Te_3_ have a triple enhancement in ideal shear strength derived from the newly formed van der Waals Te1–Te1 bond at TB, improving the interaction in Te1–Bi–Te2–Bi–Te1 five-layer substructures [57]. For InSb thermoelectric material, the shear strength of the nanotwinned structure is 2.10 GPa, which is 11% higher than that of the flawless crystal structure. The twin boundaries model is plotted in Figure 3a. The DFT [58] and molecular dynamics (MD) simulations [59] illustrate that twin boundaries play an important role in reinforcement due to the structural deformation in two sides of TB responses differently under shear, and directional covalent bond rearrangement enhances structural rigidity. While loading shear along (111)[11-2] direction, the existence of stacking-induced deformation twins originates from breaking the In-Sb directional bond, and the twins lead to a large yield stage by hindering dislocation gliding, resulting in a shear strain of ~0.85, which is more than twice than that of other brittle fracture directions. Furthermore, while TB orientation and spacing are 43.31° and 1.12 mm, the ideal strength and fracture toughness can be enhanced by 56.2% and 34.3%, as shown in Figure 3b [60]. PbTe is a brittle single crystal but becomes ductile by imposing nanotwins [61]. Different numbers and spacing of twins also have different affections. For example, with the increase of Pb-terminated coherent twin boundary (CTB) in (111) orientation, the shear stress nanotwinned PbTe decreases while the failure strain increases, indicating PbTe becomes more ductile, as shown in Figure 3d. Besides, because the strength of the Pb–Te bond in the vicinity of CTB is weakest, two halves of parts divided by TB can slip relatively without structural destruction in the yield stage. The catching bond leading the Pb–CTB to migrate to Te–CTB is the origin of the enhancement in the deformation of nanotwinned PbTe. In Cu_2_Se with liquid-like behavior, the deformation twinning evolution was observed under compression above 800 K, and the density of twins increases with temperature, resulting in different deformation modes temperatures below 600 K and above 800 K [62]. Figure 3f shows that while compressing Cu_2_Se at a higher temperature, activated shear deformation is synergistic with the compressive deformation, and the appearance of ductility benefits from twinning and Cu fluid motion. Further study on chemical bond change in deformation can improve understanding of the effect of nanotwin on enhancing the ductility of thermoelectric materials.

### 3.3. High-Entropy Engineering

**Figure 4:**
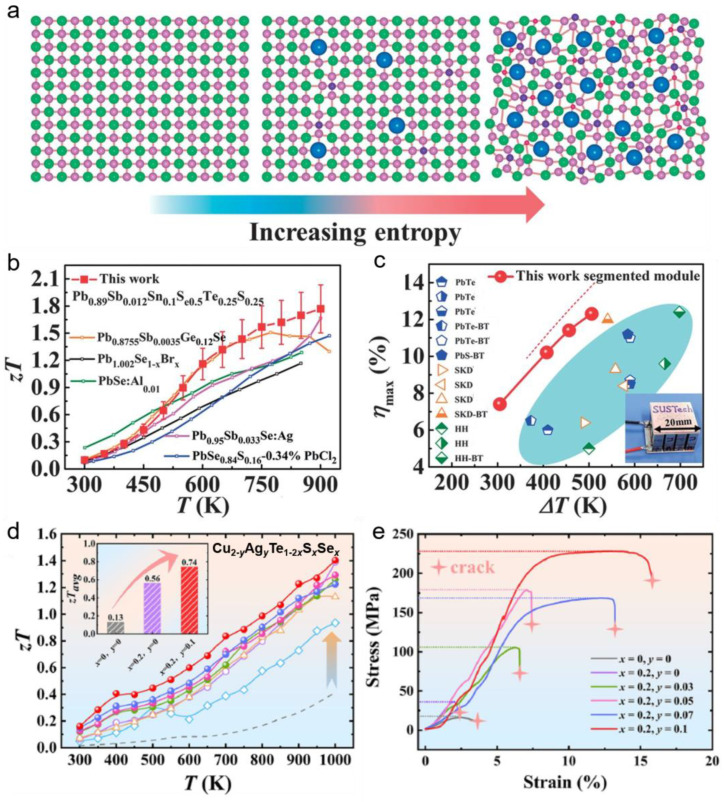
High-entropy engineering of thermoelectric materials. (**a**) Enhanced configurational entropy with increasing components in multicomponent thermoelectric materials [65]; The thermoelectric figure of merit (**b**) and conversion efficiency as a function of temperature difference (**c**) of Pb_0.89_Sb_0.012_Sn_0.1_Se_0.5_Te_0.25_S_0.25_ six-component thermoelectric materials [65] (Reprinted with permission from ref [65]. Copyright 2021 American Association for the Advancement of Science); The thermoelectric figure of merit (**d**) and stress-strain curves (**e**) of Cu_2-_*_y_*Ag*_y_*Te_1−2_*_x_*S*_x_*Se*_x_* (0 ≤ *x* ≤ 0.3, 0 ≤ *y* ≤ 0.1) multicomponent thermoelectric materials (Reprinted with permission from ref [66]. Copyright 2022 Elsevier).

Multicomponent thermoelectric materials based on “configurational entropy” are expected to surpass the thermoelectric properties of traditional thermoelectric materials. According to the definition of configurational entropy Δ*S_conf_*: $\Delta S_{conf}=-R\overset{n}{\underset{i=1}{\sum }}x_{i}\ln x_{i}$, where *x_i_* is the atomic percentage of the *i* element, and *R* is the ideal gas constant. It is easy to find that, with increasing the number of solid solution components, the configurational entropy gradually increases, as shown in Figure 4a. This kind of material containing multiple principal components is called multi-principal-element materials, also named high-entropy materials [63]. Liu et al. [64] designed a quaternary Cu_2_(S/Se/Te) multi-principal thermoelectric materials, the *zT* value of which is increased by ~50% compared with the Cu_2_Se-based materials. The thermoelectric properties of Pb_0.89_Sb_0.012_Sn_0.1_Se_0.5_Te_0.25_S_0.25_ six-component thermoelectric materials are depicted in Figure 4b,c, that *zT* value achieves 1.8 at 900 K, twice higher than that of Pb_0.99_Sb_0.012_Se material [65]. In addition, the maximum conversion efficiency is remarkable (12.3% at 507 K temperature difference), which is due to the high configuration entropy stabilizing the phase structure and electron transport capability, while the severe lattice distortion strongly scatters phonons, thereby greatly reducing the lattice thermal conductivity. “High-entropy engineering” can not only improve the thermoelectric properties of multi-principal thermoelectric materials but also improve their mechanical properties [65,66]. For example, the *zT* values of Cu_2-_*_y_*Ag*_y_*Te_1-2_*_x_*S*_x_*Se*_x_* (0 ≤ *x* ≤ 0.3, 0 ≤ *y* ≤ 0.1) multi-principal component thermoelectric materials are 2.5 times higher than that of Cu_2_Te-based material. Besides, the compressive strength is significantly increased by nearly 5 times and even induces prominent plastic deformation, which is owing to the lattice distortion hindering the movement of dislocations, showing strong solid solution strengthening [66].

### 3.4. Other Toughening Strategies

**Figure 5:**
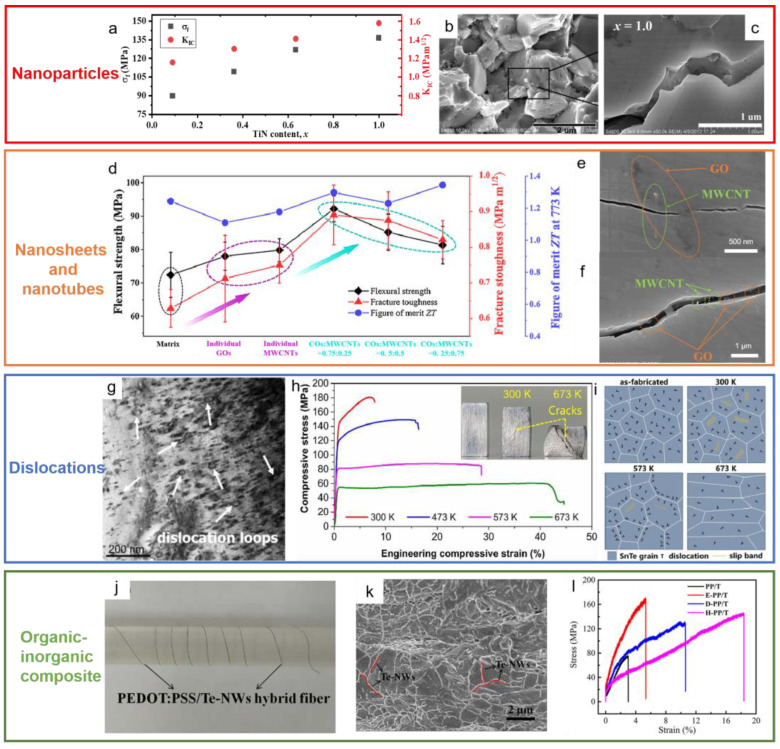
Other toughening strategies. (**a**) Flexural strength and fracture toughness of CoSb_2.875_Te_0.125_+ *x* vol.% TiN [67]; (**b**) SEM images of the sample with 1.0 vol.% TiN composites [67]; (**c**) Toughening by crack bridging, branching, and deflection (Reprinted with permission from ref [67]. Copyright 2012 Elsevier); (**d**) Mechanical and thermoelectric properties of Mg_2_(Si_0.3_Sn_0.7_)_0.99_Sb_0.01_ improved by graphene oxide nanosheets and multi-walled carbon nanotubes [68]; (**e**,**f**) Crack propagates: pulling out and crack bridging (Reprinted with permission from ref [68]. Copyright 2021 Elsevier); (**g**) Dense dislocations loops in SnTe [69]; (**h**) Stress-strain curves for compression tests of SnTe at different temperature [69]; (**i**) The deformation mechanisms in SnTe samples at different temperatures (Reprinted with permission from ref [69]. Copyright 2023 Elsevier); (**j**) The digital photograph of PEDOT:PSS/Te–NWs (PP/T) hybrid fibers [70]; (**k**) SEM images of PP/T hybrid fibers [70]; (**l**) Stress-strain curves of PP/T before and after post-treatment. (Reprinted with permission from ref [70] Copyright 2021 Elsevier).

However, few above-mentioned bulk thermoelectric materials with large deformability are usually based on specific single-crystal systems or limited by complicated preparation processes. It has been found that the composite of nano-precipitates is an effective method to improve the mechanical properties of thermoelectric materials. Duan et al. [67] dispersed 1.0 vol.% nano-TiN to CoSb_2.875_Te_0.125_ and found that compared with the matrix materials, its flexural strength and fracture toughness increased by 31% and 40%, respectively, as shown in Figure 5a. Huang et al. [68] added graphene oxide nanosheets and multi-walled carbon nanotubes together to Mg_2_(Si_0.3_Sn_0.7_)_0.99_Sb_0.01_ to prepare a bulk composite thermoelectric material, which significantly improved the thermoelectric and mechanical properties of the matrix material. In addition, Figure 5d reveals dislocations have a positive effect on ductility enhancement. Yang et al. [69] performed uniaxial compression tests on SnTe at different temperatures ranging from room temperature to 673K and found that the engineering compressive strain increased dramatically from 7.5% to 42%. Through TEM and first-principle calculations, it is found that there are considerable pre-existing dislocations in the grains of SnTe after preparation, as shown in Figure 5g. During the compression test at room temperature, the pre-existing dislocations move locally and from slip bands leading the large deformability. Due to thermal activation, Figure 5i exhibits that the pre-existing dislocations move from the grain inner to the grain boundary with the temperature rise. Such a long path for dislocation migration is the main reason for the better deformability at higher temperatures, as shown in Figure 5h. The organic/inorganic composite is another approach to enhance mechanical performance. As shown in Figure 5j–l, Liu et al. [70] composed organic/inorganic hybrid fibers with poly(3,4-ethylenedioxythiophene):poly(styrenesulfonate) (PEDOT:PSS) and Te nanowires, which exhibit a six-fold improvement of the elongation at break for post-treated hybrid fibers compared to those before treatment. Philip et al. [71] found that the flexibility of *ε–*Fe_2_O_3–_terephalate thin film increases with increased organic content while the critical bending radius reduces to 1/3.

## 4. Flexible Thermoelectric Devices

**Figure 6:**
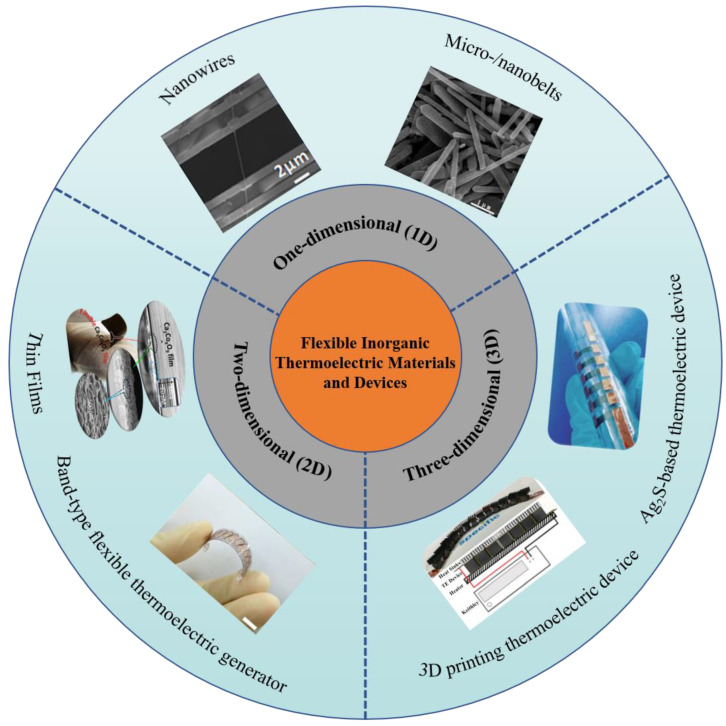
A schematic diagram of flexible thermoelectric materials and devices, including one-dimensional nanowires (Reprinted with permission from ref [76]. Copyright 2012 American Chemical Society) and micro-/nanobelts (Reprinted with permission from ref [77] Copyright 2017 Elsevier); Two-dimensional band-type flexible thermoelectric generators (Reprinted with permission from ref [78]. Copyright 2014 Royal Society of Chemistry), and thin film (Reprinted with permission from ref [79]. Copyright 2017 American Chemical Society); Three-dimensional Ag_2_S-based thermoelectric material (Reprinted with permission from ref [80]. Copyright 2019 Royal Society of Chemistry) and 3D printing thermoelectric devices (Reprinted from ref [81] under the terms of a Creative Commons CC BY License. Publish 2019 John Wiley and Sons).

Flexible thermoelectric devices take advantage of no noise, high reliability, easy miniaturization, and long service life, especially the certain flexibility in shape, which makes them good candidates for wearable electronic devices used in power generation and refrigeration [8,72,73,74,75]. Here, we concluded the design of flexible thermoelectric materials and devices into three parts: one-dimensional (1D), two-dimensional (2D), and three-dimensional (3D) flexible thermoelectric materials and devices. For example, one-dimensional nanowires [76] and micro-/nanobelts [77]; two-dimensional band-type flexible thermoelectric generator [78], and thin film [79]; three-dimensional Ag_2_S-based thermoelectric material [80] and 3D printing thermoelectric devices [81], as plotted in Figure 6.

**Figure 7:**
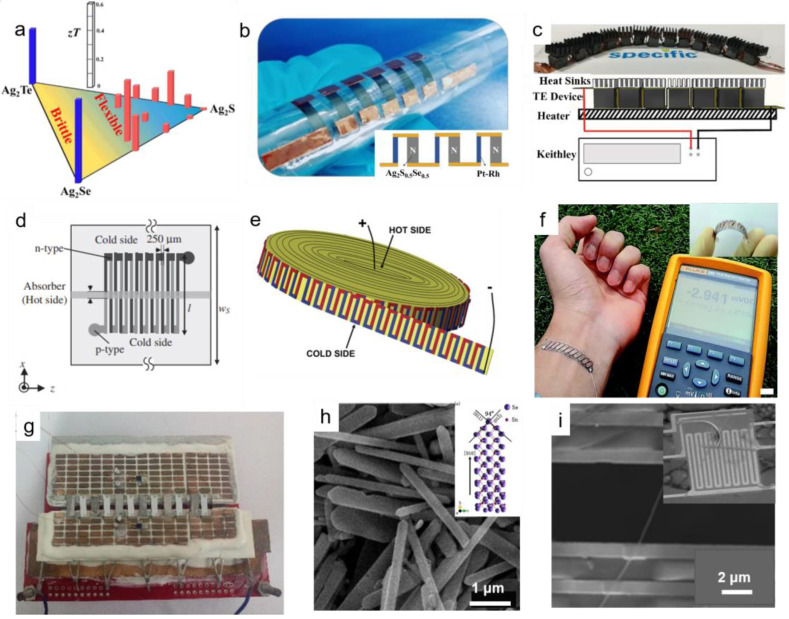
Flexible thermoelectric materials and devices with different dimensions. (**a**) The flexibility-*zT* phase diagram of Ag_2_Se, Ag_2_Te, and Ag_2_S systems [80]; (**b**) The six-couple Ag_2_S_0.5_Se_0.5_/Pt-Rh in-plane thermoelectric device and the schematic in the inset (Reprinted with permission from ref [80]. Copyright 2019 Royal Society of Chemistry); (**c**) The fabrication of the 3D printed chain-like SnSe thermoelectric devices and the schematic illustration of device performance (Reprinted from ref [81] under the terms of a Creative Commons CC BY License. Publish 2019 John Wiley and Sons); (**d**) The thermoelectric thin film modules for thermal-photovoltaic hybrid solar generator (Reprinted with permission from ref [97]. Copyright 2012 The Japan Society of Applied Physics). (**e**) Bi_0.85_Sb_0.15_-alloy screen-printing pastes on a thin polyimide foil (Reprinted with permission from ref [98]. Copyright 2006 Elsevier); (**f**) The band-type flexible thermoelectric generator and the application of harvesting thermal energy from human skin (Reprinted with permission from ref [78]. Copyright 2014 Royal Society of Chemistry); (**g**) A thermoelectric device based on organic/inorganic composite thin film (Reprinted with permission from ref [99] Copyright 2017 Elsevier) (**h**) The SEM image and the crystallographic atomic arrangement of the high-quality SnSe single crystalline nanobelts (Reprinted with permission from ref [77] Copyright 2017 Elsevier); (**i**) The thermoelectric measurement of the SiGe nanowires, the low-magnification figure as shown in the inset (Reprinted with permission from ref [76]. Copyright 2012 American Chemical Society).

Up to now, many studies have reported that the thermoelectric devices designed by bulk possess a high energy conversion efficiency. For example, Eom et al. [82] fabricated thermoelectric legs by mechanical manufacturing such as slicing and drilling. After electroplating nickel layers onto TE elements, the device was assembled and soldered with solder paste. Employing the one-step hot press technique, the single Mg_3_Sb_2_-based thermoelectric leg which fabricated with contact layers on both ends, and the measured maximum conversion efficiency is 10.6% at 400 °C [83]. He et al. [84] realized a high *zT* value of 1.7 at 750 K in non-nanostructure S-doped n-type PbTe thermoelectric material and high conversion efficiencies of up to 12.2%. Cao et al. [85] achieved an extremely high power conversion efficiency of 13.6% between 280 K and 773 K in segmented Bi_0.5_Sb_1.5_Te_3_–GeTe thermoelectric. With the increase in conversion efficiency, thermoelectric materials of different types and construction can be applied in heating, refrigeration, and power generation field [86,87,88]. Otherwise, the development of flexible 3D thermoelectric materials and devices enriched the application range and promoted the commercial use of thermoelectric materials. The Ag_2_S-based thermoelectric materials, with the ductile feature, exhibit good carrier mobility, outstanding powder factor, and a high *zT* value of 0.63 at 450 K [80]. Figure 7a shows the flexibility-*zT* phase diagram of Ag_2_Se, Ag_2_Te, and Ag_2_S systems, and Figure 7b shows the mechanical flexibility of Ag_2_S_0.5_Se_0.5_ and Ag_2_S_0.8_Te_0.2_ samples. The schematic of the six-couple Ag_2_S_0.5_Se_0.5_/Pt-Rh in-plane thermoelectric device with a high normalized maximum power density of 0.08 W/m^2^. This work provided a huge distribution to the development of bendable sensors and electronics. Matthew et al. [81] reported a novel pseudo-3D printing technique to fabricate SnSe thermoelectric devices. Figure 7c shows the fabrication of the 3D printing chain-like SnSe thermoelectric devices and the schematic illustration of device performance. The SnSe ink was acquired by ball milling, binder mixing, and curing. Then, the 3D printed mask in which Cu tape was placed to allow a Z-type series connection, and a simple p-type thermoelectric generator was fabricated by infiltrating the mask with the SnSe ink. After the ink and mask dried at 120 °C in air, cut away the mask and attach the aluminum heat sinks (14 mm × 14 mm × 7 mm) to the top of each leg. The high *zT* value of 1.7, the simple and quick preparation process, and the mobility feature make this thermoelectric generator possess a large commercial value. Moreover, thermal spray and laser micromachining eliminate the need for adhesive or mechanical bonding. Tewolde et al. [89] applied the thermal spray technique to fabricate dielectric layers, element interconnects, and thermoelectric legs. Sequentially, the electrically isolated features were patterned by laser micromachining.

Low-dimensional thermoelectric materials show improved thermoelectric properties, as mentioned in Section 3.1 [90,91]. Since 1980, considerable studies have confirmed that the nanometer size effect and quantum effect in film can significantly improve the Seebeck coefficient and reduce the thermal conductivity of thermoelectric materials, and lots of corresponding theoretical models have been proposed [92,93]. The main reason for the improvement of the *zT* value is that the material can produce a quantum confinement effect after low-dimensionalization, which limits the electron motion. Near the Fermi level, the increase in the density of states will significantly increase the Seebeck coefficient of low-dimensional thermoelectric materials. Secondly, low-dimensional materials have more grain boundaries that can scatter phonons than bulk materials, which can greatly reduce the lattice thermal conductivity, and the scattering effect of disordered atoms on the grain boundaries will also affect the electrical properties of the films [94].

In this case, two-dimensional (2D) flexible thermoelectric materials such as films, nano-and microplates, and nanowires exhibit better thermoelectric properties and wider application scenarios due to the advantages of lightweight, small size, easy bending, and cuttable. 2D TE devices are generally fabricated by inorganic TE materials sputter-deposition [79], screening printing [78], inkjet printing [95], and dispenser printing [96] on organic flexible substrates. Paul et al. [79] developed a nanostructure Ca_3_Co_4_O_9_ flexible film on mica by reactive magnetron co-sputtering. The excellent toughness makes the Ca_3_Co_4_O_9_ film with a bending radius of 14 mm, and without deteriorating the thermoelectric properties, leading to the highest thermoelectric powder factor reach 1×10^−4^ W m^−1^ K^−2^ near 300 °C. The high power factor and mechanical flexibility make the film present a wide application potential in the area of flexible thermoelectric [79]. Similarly, Mizoshiri et al. [97] prepared Bi_0.5_Sb_1.5_Te_3_ (p-type) and Bi_2_Te_2.7_Se_0.3_ (n-type) thermoelectric thin films by radio-frequency magnetron sputtering method and fabricated thin-film thermoelectric modules for the thermal-photovoltaic hybrid solar generator, as shown in Figure 7d. This work enables a 1.3 % increase in the total open voltage of the thermophotovoltaic hybrid generator compared to individual PV modules. Weber et al. [98] developed Bi_0.85_Sb_0.15_-alloy screen-printing pastes on a thin polyimide foil, as shown in Figure 7e, and the printed thermo-couples showed a good thermopower with an acceptable electrical resistance. This method provides one possibility to deposit hundred-fold thicker thermoelectric films. Converting body heat into electrical energy through thermoelectric generators to create wearable, self-powered mobile electronic systems for use in medical sensors or smart watches has become a research hotspot. Based on a flexible thermoelectric generator made of glass fabric, Sun et al. [78] fabricated a flexible thermoelectric generator using screen printing technology and a self-sustaining structure of a thermoelectric device without a top and bottom substrate. Figure 7f shows the band-type flexible thermoelectric generator and the application of harvesting thermal energy from human skin. This band-type flexible glass fabric thermoelectric generator consists of 11 thermoelectric couples and can generate 2.9 mV open-circuit output voltage and 3 μW output power on a matched external load at an air temperature of 15 °C. The generated power density can support activating some portable microwatt electronic devices. Lu et al. [95] formulated the synthesized nanoparticles into aqueous inkjet and printed the thermoelectric legs and electrodes on polyimide to fabricate the flexible thin film device. Employing the dispenser, Chien et al. [96] manufactured a planar TEG whose output power can reach 68 μW at a temperature difference of 33 K and remained stable after 500 bending cycles. As shown in Figure 7g, Li et al. [99] assembled a thermoelectric thin film device with Te nanowires and PEDOT:PSS. The flexible composite thin film keeps a stable electrical conductivity after a thousand bending cycles and exhibits a high power density value and output voltage. Zhang et al. [100] prepared the nanohybrids filled polymer composites by integrating reduced graphene oxide (rGO) and fluorinated C_60_ (F-C_60_) nanohybrids to PEDOT:PSS, a significant 19-fold enhancement in power factor (83.2 μW/m K^2^) compared to that of PEDOT:PSS (4.38 μW/m K^2^).

Generally, one-dimensional materials include quantum wires, nanowires, and nanoplates. Quantum wires like GaAs are fabricated by molecular beam epitaxy (MBE) of (AlGa)As on substrates [101], focused ion beam implantation, electron beam lithography, and dry etching [102]. For example, Nötzel et al. [101] fabricated three-dimensional arrays of quantum wires on GaAs substrates by molecular beam epitaxy. High-quality SnSe single crystalline nanobelts were prepared by solvothermal technique, and the *zT* value of which along the in-plane direction at 803 K is around 0.83, which is 60% higher than that of the previous corresponding polycrystalline samples [77]. Nanowires are grown by vapor-liquid-solid (VLS) [76], chemical vapor deposition (CVD) [103], and electrodeposition [104] on glass/nanoporous alumina membranes. For instance, The SEM image and the crystallographic atomic arrangement are shown in Figure 7h. Reducing thermal conductivity by suppressing phonon transport is an effective way to increase *zT*. Based on this method, The Si–Ge alloy and its nanowire structure were grown by the VLS method to maximize consumed the heat-carrying phonon and found that when the nanowire diameter exceeds ~100 nm, the surface boundary scattering is significant [76]. Figure 7i presents the morphology of Si-Ge nanowires and the microdevice assembly, showing that the *zT* value of Si-Ge nanowires is 0.46 at 450 K and 2.2 at 800 K, which is greatly improved compared with the bulk with the same composition.

**Figure 8:**
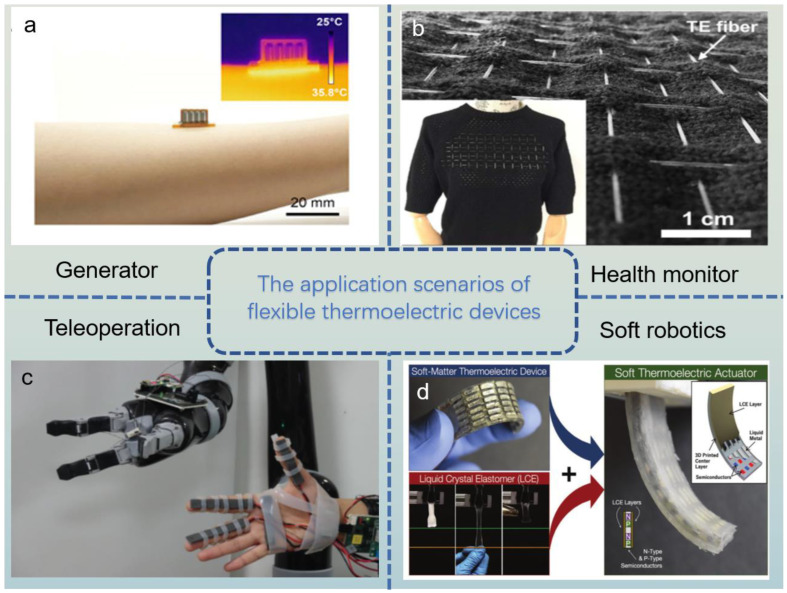
A schematic diagram of application scenarios of flexible thermoelectric devices. (**a**) Generator (Reprinted with permission from ref [109] under the terms of a Creative Commons CC BY-NC License. Publish 2021 American Association for the Advancement of Science); (**b**) Health monitor (Reprinted with permission from ref [110]. Copyright 2017 Elsevier); (**c**) Teleoperation (Reprinted with permission from ref [113] under the terms of a Creative Commons CC BY License. Publish 2021 IEEE); (**d**) Soft robotics (Reprinted with permission from ref [114] under the terms of a Creative Commons CC BY-NC License. Publish 2022 Wiley-VCH GmbH).

Given the small size, high reliability, bending, and self-powered merits, the flexible thermoelectric materials show great research potential and application scenarios in the human generator, health monitor, teleoperation, and soft robotics, as shown in Figure 8 [105,106,107,108]. For example, Ren et al. [109] designed a wearable thermoelectric generator with excellent mechanical properties that can be bent, stretched, and even worn on a finger. Besides, in the medical field, Zhang et al. [110] constructed thermoelectric clothes that can acquire thermal energy from human body heat and monitor the body temperature. Torfs et al. [111] designed an energy-autonomous wireless pulse device for a health monitor, using temperature differences to generate electricity for the device. Wang et al. [112] designed a wearable self-powered sensing system composed of Bi_2_Te_3_-based thin film and a flexible polymer pressure sensor with high thermal conductivity. A 78 mV open voltage and 7.9 μW power are obtained when the temperature gradient is 20 K in the device, and the internal resistance hardly changes after the device goes through thousands of bending cycles, which can be used in health monitoring. Lee et al. [113] assembled a thermal feedback remote control operating system. The operator can control the robot’s hand and feel the temperature of the contact object. Zadan et al. [114] devised an actuator of the soft robot with TE element and liquid crystal elastomers, which can move toward a heat source and absorb energy. Through the Seebeck effect, TEG can convert thermal energy directly into electrical energy [115]. Based on this, the concepts of solar thermoelectric generators and photovoltaic-thermoelectric hybrid systems are proposed. Li et al. established a solar thermoelectric generator model to predict the conversion efficiency of different thermoelectric materials and concentration ratios and found that it reached high system efficiency (~14%) at the concentration ratio of skutterudite around 400 [105]. Furthermore, as the size of electronic and bioanalytical equipment gradually decreases, the size requirements for electronic packages and chip heat dissipation are higher [116]. Thermoelectric coolers made of nanostructured Bi_2_Te_3_-based thin film are integrated on a silicon chip and reach a high heat flux by passing a ~3A DC [87]. In addition, rigid thermoelectrical elements and non-stretchable substrates are assembled into flexible thermoelectrical devices, which deform by stretching and bending and result in a deformation ratio of 20% [117]. As the device size decreases to micro-/nano-sizes [118], there are more harsh requirements for the flexibility, portability, and even wearability of thermoelectric devices. With the research developed in-depth, it is believed that flexible thermoelectric materials will be widely used in our daily life, providing more convenient living conditions for human beings and effective energy recovery for the global environment.

## 5. Conclusions

In this review, we summarized recent advances in ductile thermoelectric materials and their applications in three sections:

(1) The intrinsic ductility of thermoelectric materials like *α–*Ag_2_S, *β–*InSe, and SnSe_2_ can be achieved by optimal configurations of chemical bonds and crystal structures. The deformability factor criterion is expected to develop more intrinsically ductile thermoelectric materials.
(2) To further improve the mechanical properties of thermoelectric materials, we summarized promising toughness strategies, including the size effect, twin boundary engineering, and high-entropy engineering. We also briefly concluded some other toughening strategies, i.e., nano-precipitates, dislocation engineering, and temperature effect.
(3) With the recent advances in ductile thermoelectric materials with high thermoelectric performance, flexible thermoelectric devices have been designed and fabricated with different dimensions to cater to various application scenarios, including generators, health monitors, teleoperation, and soft robotics.

## Data Availability

The raw data required to reproduce these findings are available upon reasonable request.
